# Effect of different acupuncture and moxibustion methods on functional dyspepsia caused by sequelae of COVID-19: A protocol for systematic review and meta-analysis

**DOI:** 10.1097/MD.0000000000030770

**Published:** 2022-09-23

**Authors:** Tianzhong Peng, Xuedi Huang, Manhua Zhu, Xinju Hou, Yue Xiong, Xinyue Fang, Zitong Lin, Lu Liu, Wanning Lan, Xingzhen Lin

**Affiliations:** a Nanchang Hongdu Hospital of Traditional Chinese Medicine, Jiangxi, China; b Jiangxi Province Hospital of Integrated Chinese Western Medicine, Jiangxi, China; c Guangzhou University of Chinese Medicine, Guangzhou, China.

**Keywords:** acupuncture and moxibustion, COVID-19, functional dyspepsia, protocol, systematic review

## Abstract

Functional dyspepsia (FD) is a group of diseases that cannot be explained after routine clinical examination, and is characterized by postprandial fullness, early satiety, and upper abdominal pain or burning. According to the statistics, FD continues to become one of the high-risk sequelae of coronavirus disease 2019 (COVID-19), affecting patients’ quality of life, increasing psychological burden and increasing economic costs. However, its optimal treatment is still an urgent problem. A large number of studies have shown that acupuncture and moxibustion is effective and safe in the treatment of FD caused by sequelae of COVID-19, which is of research value. Therefore, based on the current literatures, the effectiveness and safety of different acupuncture and moxibustion methods were systematically evaluated to provide possible alternative therapy on FD.

**Methods:**

Studies search for eligible randomized controlled trials that use different acupuncture and moxibustion methods as the sole treatment on FD and their data extraction will be done by 2 researchers. In case of disagreement, a third researcher will be introduced for arbitration. Mean difference or relative risk with fixed or random effect model in terms of 95% confidence interval will be adopted for the data synthesis. To evaluate the risk of bias, the Cochrane risk of bias assessment tool will be utilized. The sensitivity or subgroup analysis will also be conducted when meeting high heterogeneity (*I*^2^ > 50%).

**Results:**

This meta-analysis will provide an authentic synthesis of different acupuncture and moxibustion methods on FD caused by sequelae of COVID-19.

**Conclusion:**

This meta-analysis will evaluate the effect of acupuncture and moxibustion on FD caused by sequelae of COVID-19, providing evidence as to the treatment in these patients.

## 1. Introduction

Coronavirus disease 2019 (COVID-19) caused by the SARS-CoV-2 has caused extremely serious harm to human’s life and health since the outbreak at the end of 2019. With the number of healers of COVID-19 is increasing, the emergence of long-term persistent symptoms has become another focus of attention for experts from all countries after the acute infection period. A large number of early studies have confirmed that patients with COVID-19 have clinical manifestations of functional dyspepsia (FD) during infection such as loss of appetite, diarrhea, vomiting and other gastrointestinal symptoms.^[1]^ The frequency of digestive symptoms in patients with COVID-19 presents regional differently, for higher in North America, followed by Europe and Asia.^[2,3]^ FD is a digestive system disease characterized by epigastric symptoms, including 2 major types of etiology: organic and functional. FD’s clinical manifestations are mostly postprandial abdominal bloating and discomfort, early satiety, epigastric pain, vomiting, nausea and belching.^[4]^ At present, the diagnosis of FD is mainly based on the latest Rome IV standard for functional gastrointestinal diseases, which was formulated by the Rome Committee in 2016. Compared with the Rome III standard, the Rome IV standard has adjusted in terms of the frequency, severity and subgroup diagnosis of specific symptoms.^[5]^ The diagnosis of FD still requires >6 months’ disease process, as well as the symptoms should having been onset in the past 3 months. In addition, the extent of 4 core symptoms (epigastric pain, early satiety, postprandial discomfort and epigastric burning) was assessed to the degree of symptoms reached an uncomfortable level affecting daily life.^[6]^ The overall global prevalence of FD is 11.5% to 14.5%,^[7]^ at the same time rising in recent years. Up to now, the etiology and pathogenesis of FD are still unclear, which bring great difficulties to clinical treatment, resulting in repeated medical treatment, seriously affecting the quality of life and consuming a lot of medical resources. Therefore, it is necessary to seek effective treatment.

At present, there have been many clinical research of the use of gastrointestinal motility drugs on FD, but still without uniformity and consensus. Conventional therapies include gastro-kinetic agents, acid suppressive drugs, drugs for eradication of helicobacter pylori (HP) infection, and antidepressants. Gastro-kinetic agent is dopamine receptor antagonists such as domperidone and itopride, 5-HT4 receptor agonists such as levosulpiride and mosapride. Acid suppressive drugs mainly include H2 receptor antagonists, and proton pump inhibitors. Antidepressants are doxepin and flunarizine, etc. However, due to the unclear mechanism of gastrointestinal symptoms after COVID-19, conventional therapies such as gastro-kinetic agents are currently used.

In this situation, acupuncture and moxibustion therapies increasingly appear in clinical treatment of FD.^[8]^ The therapies are diverse, safe and effective, which could effectively alleviate the clinical symptoms of FD’s patients. Acupuncture and moxibustion therapy has a variety of forms and methods, such as ordinary filiform needle acupuncture, abdominal needle, warm acupuncture, electro-acupuncture, mild moxibustion, thermo-sensitive moxibustion, etc., having a good effect on FD and other gastrointestinal diseases.

To sum up, the vague pathogenesis of FD caused by COVID-19 and unsystematic treatment resulting in the lack of supporting systematic evidence, which has had a negative impact of the treatment to a certain extent. However, acupuncture and moxibustion therapy of highly effective, is only regarded as supplementary medicine and alternative medicine. Therefore, in order to objectively understand the efficacy and safety of this oriental therapy in the treatment of FD, this study aims to collect randomized controlled trials of different methods of acupuncture and moxibustion on FD and complete systematic reviews and meta-analyses, providing a reliable evidence-based basis for clinical application.

## 2. Methods

This study has been registered as PROSPERO CRD42022346782 (https://www.crd.york.ac.uk/prospero/). This protocol for meta-analysis will be performed according to the Preferred Reporting Items for Systematic Review and Meta analysis Protocols statement.^[9]^

### 2.1. Selection criteria

#### 2.1.1. Types of studies.

There are not any limitations on the publication language or publication time. Only randomized controlled trials (RCTs) will be included in our literature, which means nonrandomized controlled trial, reviews, case reports, experimental study, observational cohort, case control studies and animal study will be deleted by our researchers.

#### 2.1.2. Participants.

COVID-19 patients with FD lasted for ≥1 week. There are no restrictions on gender, race, and stage of disease. Patients with a history of FD before COVID-19 infection will be excluded. The diagnosis of COVID-19 includes Chinese or international diagnostic criteria.

#### 2.1.3. Types of interventions.

In addition to the treatment of COVID-19, treatment group interventions comprised different methods of acupuncture and moxibustion. As well as treating COVID-19, the comparator groups intervention included comfort therapy (placebo, pseudo-acupuncture, or blank control), other therapies (Western medicine, usual care or non-drug therapy, etc).

### 2.2. Outcomes

The primary outcome will be effectiveness including the quantity of duodenum eosinophils, the status of vagus nerve and intestinal flora. The secondary outcomes will be gastrointestinal symptoms, health related quality of life, and mortality and hospitalization rates.

### 2.3. Search strategy

First, PubMed, Embase, Web of Science, Chinese National Knowledge Infrastructure database, Chinese Biomedical Database, Chinese Science and Technology Periodical database, the WanFang database and the Cochrane Central Register of Controlled Trials databases will be searched to find relevant articles up to July 2022 using a combination of the main search terms “Functional Dyspepsia,” “duodenum eosinophils,” “vagus nerve,” and “intestinal flora” within the restriction limit of “randomized controlled trial.” Taking PubMed as an example, the retrieval strategy is shown in Table 1.

**Table 1:** Search strategy for the PubMed database

Number	Search items
#1	(“post-acute COVID-19 syndrome”[Mesh]) OR (“long-COVID”[Title/Abstract])) OR (“long-haul COVID”[Title/Abstract])) OR (“post-acute COVID syndrome”[Title/Abstract])) OR (“persistent COVID-19”[Title/Abstract])) OR (“post-acute COVID19 syndrome”[Title/Abstract])) OR (“long hauler COVID”[Title/Abstract])) OR (“long COVID”[Title/Abstract])) OR (“post-acute sequelae of SARS-CoV-2 infection”[Title/Abstract])) OR (“long haul COVID”[Title/Abstract])) OR (“chronic COVID syndrome”[Title/Abstract])
#2	(“Dyspepsia”[Mesh]) OR (“Dyspepsias”[Title/Abstract]))OR (“Indigestion”[Title/Abstract])) OR (“Indigestions”[Title/Abstract])
#3	(“Acupuncture Therapy”[Mesh]) OR (“Acupuncture Treatment”[Title/Abstract])) OR (“Acupuncture Treatments”[Title/Abstract])) OR (“Treatment, Acupuncture”[Title/Abstract])) OR (“Therapy, Acupuncture”[Title/Abstract])) OR (Pharmacoacupuncture Treatment”[Title/Abstract])) OR (“Treatment, Pharmacoacupuncture”[Title/Abstract])) OR (“Pharmacoacupuncture Therapy”[Title/Abstract])) OR (“Therapy, Pharmacoacupuncture”[Title/Abstract])) OR (“Acupotomy”[Title/Abstract])) OR (“Acupotomies”[Title/Abstract])
#4	(moxibustion[Mesh]) OR (moxabustion[Title/Abstract])
#5	Randomized Controlled Trial[Filter]
#6	#1 and #2 and #3 and #4

Second, to include complete and updated outcomes, abstracts and presentations of ongoing RCTs on FD from several of the most important international conferences (American Gastroenterological Association) in 2018 to 2022 will be inspected.

Last, the reference lists of the relevant articles will be checked with expectation for additional articles.

The searching process will be conducted systematically by 2 researchers through 8 databases from their inception to the present date. When meeting with divergence, discussion with a third researcher will be conducted.

### 2.4. Study selection

**Figure 1.**
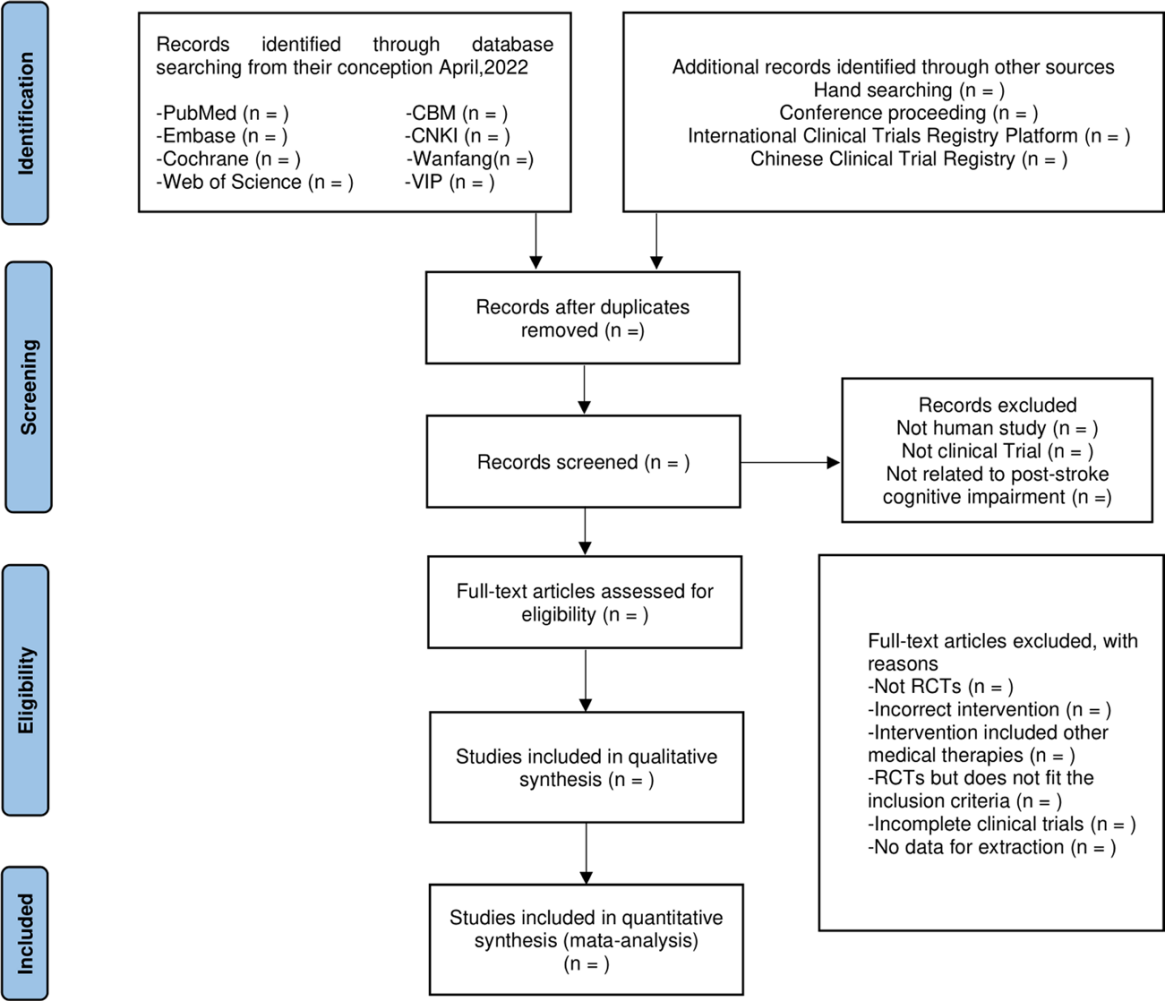
PRISMA flow chart of study selection process. PRISMA=Preferred Reporting Items for Systematic Review and Meta Analysis.

In order to screen all studies, we will use the EndNote X9 to facilitate document management. Data collection and analysis will be conducted by 2 professionally trained researchers. If there are differences, they will solve the problem through discussion or ask the opinions of a third researcher. The article will be initially screened by reviewing the title and abstract of the study’s, and then read the full text to exclude the unqualified documents: not RCTs, incorrect intervention, it belongs to RCTs, but does not meet the inclusion criteria, no data for extraction. Finally, the literature meeting the requirements will be evaluated and included in our study. The detailed screening process is shown in Figure 1.

### 2.5. Data extraction

Two reviewers will be responsible for the extraction and management of data according to the retrieval strategy, including study title, journal, year of publication, name of first author, general information, study design, experimental intervention and timing of intervention, results, and adverse events. If there is any disagreement between the 2 reviewers during the data extraction process, the panel will jointly arbitrate and make a decision.

### 2.6. Risk of bias and quality assessment

As for risk of bias assessment, we will use the tool, Cochrane System Reviewer Manual (Version 6.3, 2022) to evaluate the quality of the included RCTs.^[10]^ The quality of evidence for the outcomes will be evaluated by the use of the Grading of Recommendations Assessment, Development and Evaluation system.^[11]^

Two researchers will perform the analysis and the results will be cross-checked. We will evaluate the following 6 aspects: Selection bias: Bias arising from the randomization process; Implementation bias: Bias due to deviations from intended interventions; Measurement bias: Bias in measurement of the outcome; Follow-up bias: Bias due to missing outcome data; Reporting bias: Bias in selection of the reported result; Other bias.

The process of quality assessment will be conducted systematically by 2 researchers. When data meet with ambiguity, contradiction, errors or omission, discussion with a third reviewer will be conducted. So as to cope with potential divergence, the researcher will contact the corresponding author for the clarified, correct or missing data, when needed.

### 2.7. Assessment of reporting bias

Funnel plots will be conducted to evaluate reported deviations. We will use funnel plots to detect potential reporting bias. Begg and Egger test will be used to assess the symmetry of the funnel, draw and detect release deviations.

### 2.8. Statistical analysis

For continuous outcomes, the effect size for the intervention will be calculated by the difference between the means of the intervention and control groups at the end of the intervention. For morbidity and mortality, relative risk with 95% confidence interval will be calculated. For each outcome, heterogeneity will be assessed using the Cochran Q and *I*^2^ statistic; for the Cochran Q and *I*^2^ statistic, a *P* value of <.05 and *I*^2^ > 50% will be considered significant, respectively. When there is significant heterogeneity, the data will be pooled using a random-effects model; otherwise, a fixed-effects model will be used. Publication bias will be assessed graphically using a funnel plot and mathematically using Egger test. For these analyses, Comprehensive Meta Analysis Software version 2 (Biostat, Englewood, NJ) and STATA 16 software (Stata Corp LP, College Station, TX) will be used.

### 2.9. Sensitivity and subgroup analysis

Meta-regression will be used to determine whether the effect of acupuncture and moxibustion methods will be confounded by baseline clinical characteristics. Subgroup analysis stratified by route of different methods (warm acupuncture, thermo-sensitive moxibustion or electroacupuncture) will be performed.

### 2.10. Ethical issues

This meta-analysis is a literature study. Ethical approval is not required because this meta-analysis will not involve any subject directly.

## 3. Discussion

Regarding FD as an important sequelae of COVID-19,^[2]^ many literature have analyzed the necessity of treating FD, so as to make humanistic care for patients with COVID-19 further displayed. Although the effect of acupuncture and moxibustion methods in FD patients have been reported, there is still insufficient meta-analysis to support the conclusion. Moreover, according to the investigation, acupuncture and moxibustion on sequelae of COVID-19 have not yet been taken seriously. To the best of our knowledge, this is the first meta-analysis protocol about different acupuncture and moxibustion methods on FD caused by sequelae of COVID-19. The results will evaluate whether different ways of acupuncture and moxibustion are beneficial for the long time conditioning of FD patients with COVID-19, providing evidence regarding the choice of acupuncture and moxibustion in these patients.

## Author contributions

Xingzhen Lin is the guarantor of the article and will be the arbitrator when meeting disagreements. All research members participated in developing the criteria and drafting the protocol for this systematic review. TP, XH and MZ established the search strategy. XH, YX and ZL will independently accomplish the study selection, data extraction and assess the risk of bias. XF, LL and WL will perform the data syntheses. The subsequent and final versions of the protocol are critically reviewed, modified and authorized by all authors.

**Methodology:** Yue Xiong.

**Writing – original draft:** Tianzhong Peng, Xuedi Huang, Manhua Zhu, Xinyue Fang, Wanning Lan, Xingzhen Lin.

**Writing – review & editing:** Tianzhong Peng, Xuedi Huang, Manhua Zhu, Xinju Hou, Xinyue Fang, Zitong Lin, Lu Liu, Wanning Lan, Xingzhen Lin.
